# Case Report: Diagnostic challenges in differentiated-type vulvar intraepithelial neoplasia

**DOI:** 10.3389/fonc.2025.1662088

**Published:** 2025-11-25

**Authors:** Jifeng Peng, Yanyan Li, Xiaoling Sun, Jianxin Hu, Ying Bai, Hui Yang, Yan Wang, Xiaolong Gao, Peihao Yin, Yuling Dong

**Affiliations:** 1 School of Nursing and Health, Xi’an Innovation College of Yan’an University, Xi’an, Shaanxi, China; 2 Department of Pathology, Shandong Provincial Maternal and Child Health Care Hospital Affiliated to Qingdao University, Jinan, Shandong, China

**Keywords:** vulvar intraepithelial neoplasia, differentiated-type, higher risk, precancerous lesion, diagnosis

## Abstract

Differentiated-type vulvar intraepithelial neoplasia (dVIN) is typically HPV-independent and is most strongly linked to HPV-independent keratinizing squamous cell carcinoma (often well- to moderately differentiated). It commonly shows aberrant p53 and p16-negative immunophenotype. As such, dVIN carries a higher risk of progression to invasion and metastasis, tends to progress to carcinoma more rapidly, and predominantly affects older women. In this study, we report two cases of vulvar squamous cell carcinoma (VSCC) associated with dVIN, aiming to enhance awareness of its pathological and clinical features. In both cases, the patients tested negative for human papillomavirus (HPV), and the tumors presented as well-differentiated, keratinizing squamous cell carcinoma (keratinizing SCC) with lymph node metastasis. In Case 1, the initial pathological diagnosis was misinterpreted as inflammation or mild-to-moderate epithelial hyperplasia. It was only upon lymph node metastasis and further immunohistochemical review (including mutant-type p53, negative p16, and positive CK17 and SOX2) that dVIN was confirmed, suggesting a possible early microinvasive lesion that had been overlooked. In Case 2, the ulcerated, bleeding lesion was diagnosed as SCC with adjacent areas of dVIN, supported by immunohistochemical findings. These two cases highlight that although dVIN may appear histologically subtle, it can directly progress to SCC and even metastasize when overt invasion is not appreciated on the index biopsy. It may be prudent to maintain increased vigilance for persistent vulvar lesions, particularly in HPV-negative and histologically ambiguous settings. Comprehensive assessment (including immunohistochemistry) may facilitate earlier detection and more accurate diagnosis, thereby potentially improving treatment outcomes.

## Introduction

Differentiated-type vulvar intraepithelial neoplasia (dVIN) is a rare but clinically significant a high-risk precursor of vulvar squamous cell carcinoma (VSCC), classified as an HPV-independent form of VIN (1–3). Although it accounts for only 5%–10% of all VIN cases, dVIN carries a much higher risk of malignant transformation compared to HPV-associated usual-type VIN [usual-type vulvar intraepithelial neoplasia (uVIN) or high-grade squamous intraepithelial lesion (HSIL)]. It may progress to invasive VSCC over a shorter interval to invasive VSCC (4). Therefore, accurate recognition and timely diagnosis of dVIN are crucial.

dVIN primarily affects postmenopausal women, with older age at presentation than uVIN compared to uVIN. It is frequently associated with chronic vulvar conditions, especially lichen sclerosus (LS) (3). Lesions are typically unifocal and localized, presenting as a solitary white papule or macule, often a well-demarcated leukoplakic/keratotic plaque with occasional fissuring or superficial erosion. These presentations are often misinterpreted as benign inflammatory or hyperplastic conditions. Studies have shown that dVIN has a significantly higher risk of progressing to invasive SCC within 1–2 years of diagnosis and a higher postoperative recurrence rate than uVIN (5).

Histopathologically, dVIN shows surface maturation with prominent keratinization, whereas the basal/parabasal layers exhibit mild-to-moderate atypia, including enlarged nuclei, hyperchromasia, disordered maturation/stratification, and dyskeratosis (6). Other features such as hyperkeratosis, acanthosis, and prominent intercellular bridges are often present. However, because the cytologic atypia is subtle and predominantly basal/parabasal in distribution, dVIN can be difficult to distinguish from chronic inflammatory or reactive conditions, posing a diagnostic challenge (7).

Immunohistochemistry plays a critical role in improving diagnostic accuracy for dVIN. Among available markers, p53 is the most informative (8). Aberrant p53 expression patterns—including diffuse strong positivity (overexpression) or complete absence (null pattern) with intact internal controls—indicate TP53 mutation, which is commonly seen in dVIN. In contrast, p16 expression in dVIN is typically negative or focally weak, and Ki-67 labeling is usually confined to the lower one-third of the epithelium (9). This immunohistochemical profile is in sharp contrast to that of uVIN, which typically shows block-type p16 positivity, wild-type p53 pattern, and diffuse Ki-67 labeling extending through most or all epithelial layers. Recent molecular pathology studies further support these findings by identifying TP53 mutations as the most frequent genetic alteration in dVIN, aligning with its p53 immunohistochemical patterns and underscoring its high-risk biology with a propensity for rapid progression (5, 10).

In summary, dVIN is a precancerous lesion of the vulva characterized by distinct histologic features, a characteristic immunophenotype, and well-characterized molecular alterations. Due to its subtle clinical presentation and mild cytologic atypia, dVIN is frequently underdiagnosed or misdiagnosed. For clinicians and pathologists, heightened clinical–pathologic vigilance is warranted when evaluating persistent vulvar lesions, especially in HPV-negative and histologically ambiguous cases. A combined approach using histologic assessment, immunohistochemical profiling, and evaluation of clinical risk factors is essential for early detection, accurate diagnosis, and timely intervention. This report aims to enhance the understanding of dVIN by illustrating its typical pathologic and immunohistochemical features and emphasizing its differential diagnosis from chronic vulvar conditions.

## Case report

### Case 1

A woman in her 60s presented to our hospital with a 10-year history of vulvar pruritus, which had significantly worsened over the past year. She had intermittently after the onset of symptoms, though the specific drug names were unknown. Over the last year, the pruritus was accompanied by vulvar swelling and pain. In early 2024, she visited the dermatology department of a local hospital and was diagnosed with vulvar eczema. After topical treatment (details not available) failed to improve her condition, she sought care at a municipal hospital, where a vulvar biopsy was performed. Histopathological examination revealed acute and chronic inflammatory infiltrates in the tissue, with the surface squamous epithelium showing downward (bulbous) elongation of rete ridges and prominent basal/parabasal cytologic atypia. Warty carcinoma could not be ruled out.

**Figure 1:**
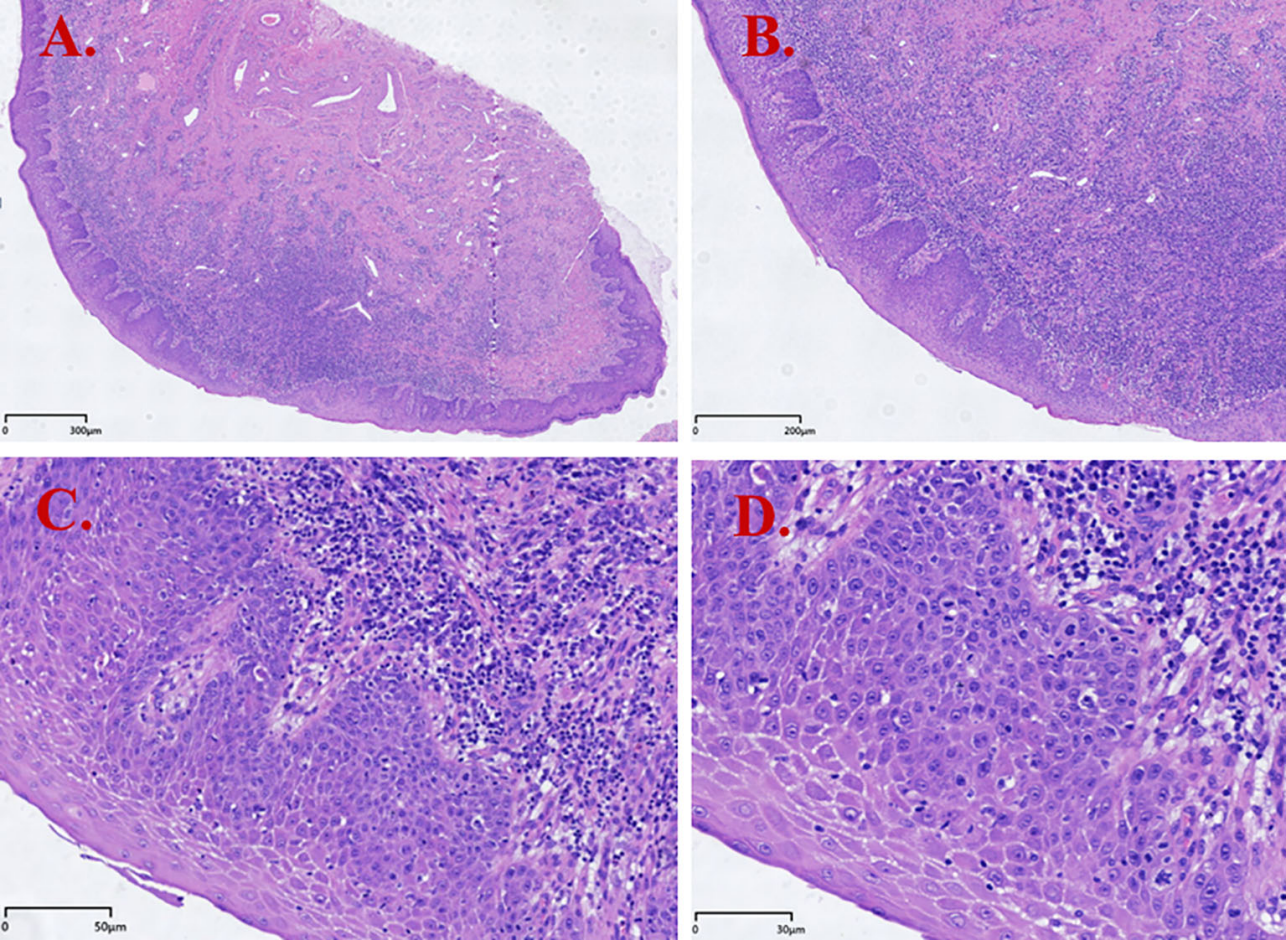
Hematoxylin and eosin (H&E) staining results of dVIN (Case 1). **(A)** Squamous epithelial hyperplasia with hyperkeratosis and parakeratosis; **(B)** Surface maturation with downward (bulbous) elongation of rete ridges; **(C)** Cells with abundant, slightly eosinophilic cytoplasm; basal cell hyperplasia; dense lymphocytic infiltration in the subepithelial stroma; **(D)** Prominent intercellular bridges and basal/parabasal cytologic atypia (enlarged nuclei with conspicuous nucleoli and vesicular/hyperchromatic chromatin).

The patient was then referred to the dermatology department of a provincial specialist hospital. Pathology consultation reported hyperkeratosis and parakeratosis of the vulvar squamous epithelium, acanthosis with basal layer hyperplasia with increased mitotic activity, focal squamous epithelial hyperplasia, and dense lymphoplasmacytic infiltration in the superficial dermis. These findings were interpreted as squamous epithelial hyperplasia with chronic inflammation (**Figure 1**). HPV testing was negative. She was treated with topical miconazole nitrate cream, fusidic acid cream, and oral levocetirizine hydrochloride tablets (5 mg once daily), resulting in slight symptom relief.

In late 2024, the patient palpated a tender mass in the right inguinal region, approximately 1 cm in diameter, but did not seek medical attention. In early 2025, the mass had increased in size to nearly 2 cm and was tender, prompting her to visit a local clinic. Ultrasonography showed a hypoechoic mass measuring 1.9×2.0×1.9 cm in the subcutaneous soft tissue of the right groin, with relatively well-defined but irregular margins. Color Doppler flow imaging (CDFI) detected a hilar vascularity; features suggestive of (reactive/necrotizing) lymphadenitis. Empiric antimicrobial therapy with intravenous cefotaxime sodium and ornidazole was ineffective.

The patient subsequently underwent a pelvic MRI at the municipal hospital, which revealed abnormal subcutaneous signal intensity in the right inguinal region, suspicious for a space-occupying lesion or enlarged lymph nodes. A biopsy of the right inguinal mass was performed, and pathology confirmed invasive, moderately differentiated SCC involving fibroadipose tissue. Immunohistochemistry showed CK5/6 (+), p40 (+), CK7 (−), Ki-67 (~40%+), CD56 (−), Syn (−), and CgA (−). Tumor marker levels were within normal ranges. She was referred to a hospital for further PET/CT evaluation, which demonstrated a well-defined soft-tissue nodule in the right groin with central low-density necrosis and high metabolic activity (SUVmax = 14.8), consistent with malignancy. Distinguishing a primary inguinal lesion from metastatic disease remained difficult. Several mildly enlarged right inguinal lymph nodes were also noted, with low-level uptake (SUVmax = 1.4).

At admission with a provisional diagnosis of vulvar malignancy, the patient reported no abdominal pain or vaginal bleeding, but had marked tenderness over the right inguinal mass on palpation. She denied fever, cough, chest pain, shortness of breath, and palpitations. Appetite, sleep, and bowel and bladder function were normal, with no significant weight change.

**Figure 2:**
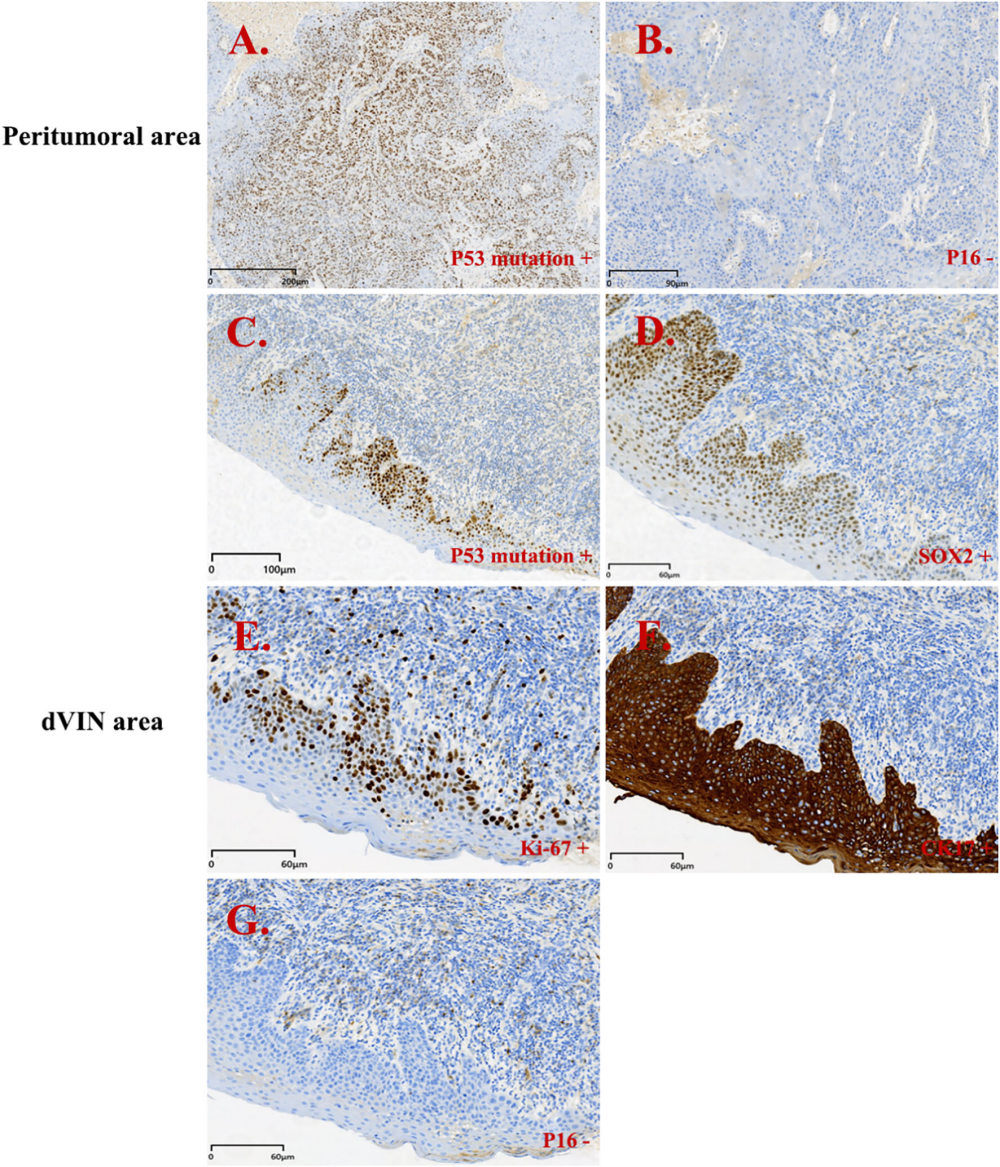
Immunohistochemistry results for patient 1. Peritumoral area (PA): **(A, B)**; dVIN area: **(C-G)**. **(A)** p53 mutation + (PA); **(B)** p16 - (PA); **(C)** p53 mutation + (dVIN); **(D)** SOX2 + (dVIN); **(E)** Ki-67 + (dVIN); **(F)** CK17 + (dVIN); **(G)** p16 - (dVIN).

She underwent partial vulvectomy and right inguinofemoral lymphadenectomy at our institution. Postoperative pathology revealed vulvar squamous epithelium with areas of hyperkeratosis and parakeratosis, thickened granular layer, acanthosis, and focal elongation of rete ridges, but no definite evidence of malignancy. Metastatic high-grade SCC involving the right superficial inguinal lymph node(s), with involvement of adjacent fibroadipose tissue. Immunohistochemical studies on the vulvar specimen demonstrated an aberrant (mutant-type) p53 pattern with strong basal nuclear overexpression, SOX2 (+), CK17 (+), and Ki-67 labeling extending to the mid-epithelial layer in some areas (**Figure 2**).

The patient subsequently received 5 cycles of the TP regimen (paclitaxel plus cisplatin) and 32 radiotherapy sessions. At 8 months post-operation, follow-up CT showed multiple nodules in the right pleural region (right shoulder and right ribs), consistent with osseous metastases.

### Case 2

A woman in her 50s presented to our hospital with a 4-month history of progressive vulvar mass enlargement accompanied by pain, occurring 6 months after surgical excision of a previous vulvar lesion. The patient reported natural menopause 3 years earlier. Two years before presentation, she noticed a firm ~1 cm nodule near the right labium minus adjacent to the clitoris, accompanied by extensive bilateral labial ulceration with bleeding, marked tenderness, pain, and pruritus; however, she did not seek medical attention. In 2024, dysuria ensued, and she underwent evaluation at a local hospital, where surgical resection of a vulvar mass was performed; per the patient’s report, the postoperative diagnosis was VSCC. Although referral to a higher-level hospital was recommended, the patient did not follow up or receive further treatment. Over the past four months, she reported that the vulvar mass had increased in size, accompanied by pain and persistent dysuria, prompting her to seek care at our hospital in early 2025. A colposcopy-guided cervical biopsy showed chronic cervicitis; however, histopathologic consultation of the prior vulvar slides at our institution confirmed well-differentiated SCC.

**Figure 3:**
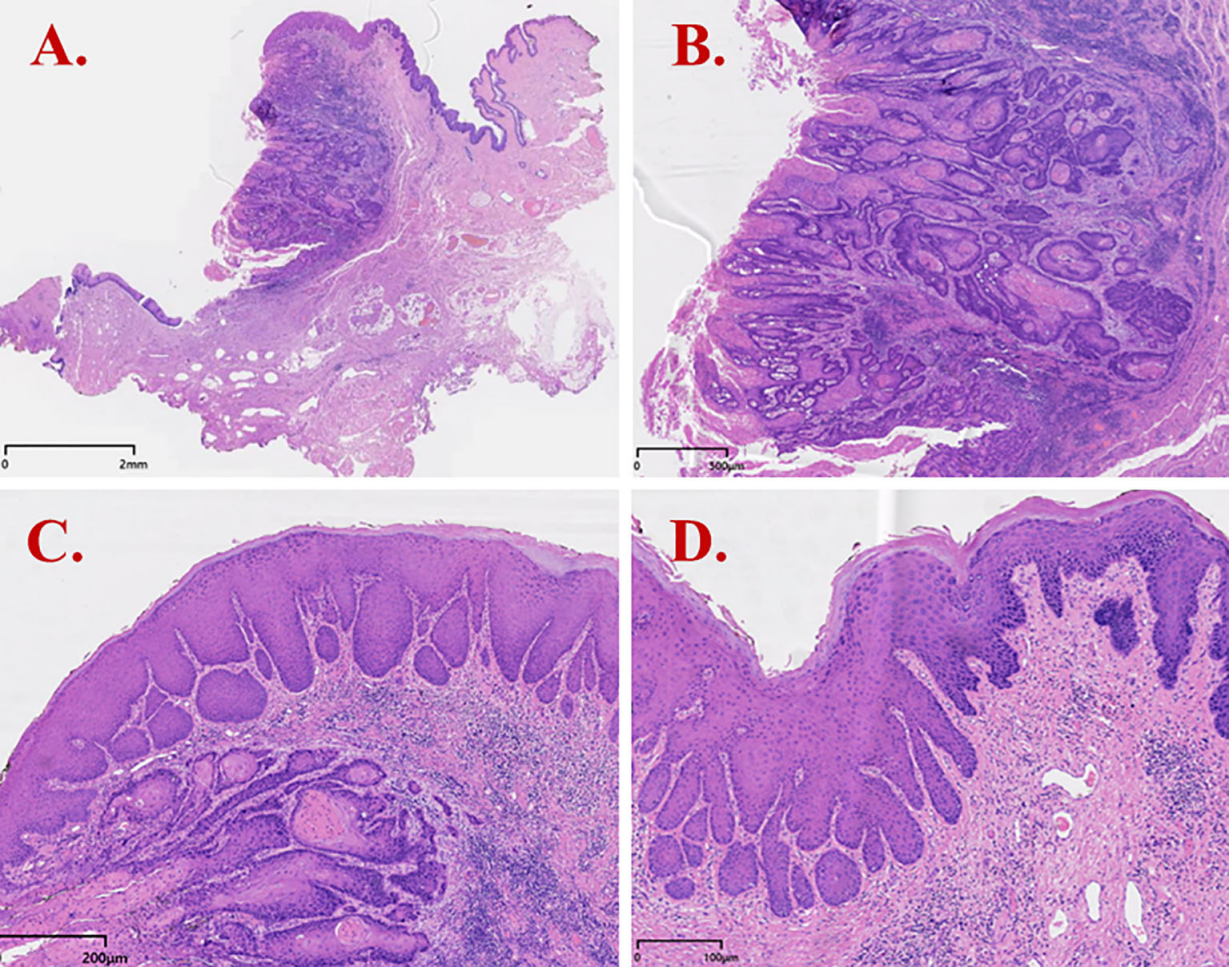
H&E staining results of dVIN (Case 2). **(A)** Invasive squamous cell carcinoma with infiltrative growth; **(B)** Tumor cells showing keratinization/maturation toward the surface and infiltrative growth with a fibromyxoid stromal reaction; **(C)** dVIN adjacent to tumor nests, with squamous cell hyperplasia, downward (bulbous) elongation of rete ridges, and preserved surface maturation; **(D)** dVIN showing cells with abundant eosinophilic cytoplasm, marked hyperkeratosis, nuclear enlargement with conspicuous nucleoli and vesicular/hyperchromatic chromatin, and prominent basal/parabasal atypia.

**Figure 4:**
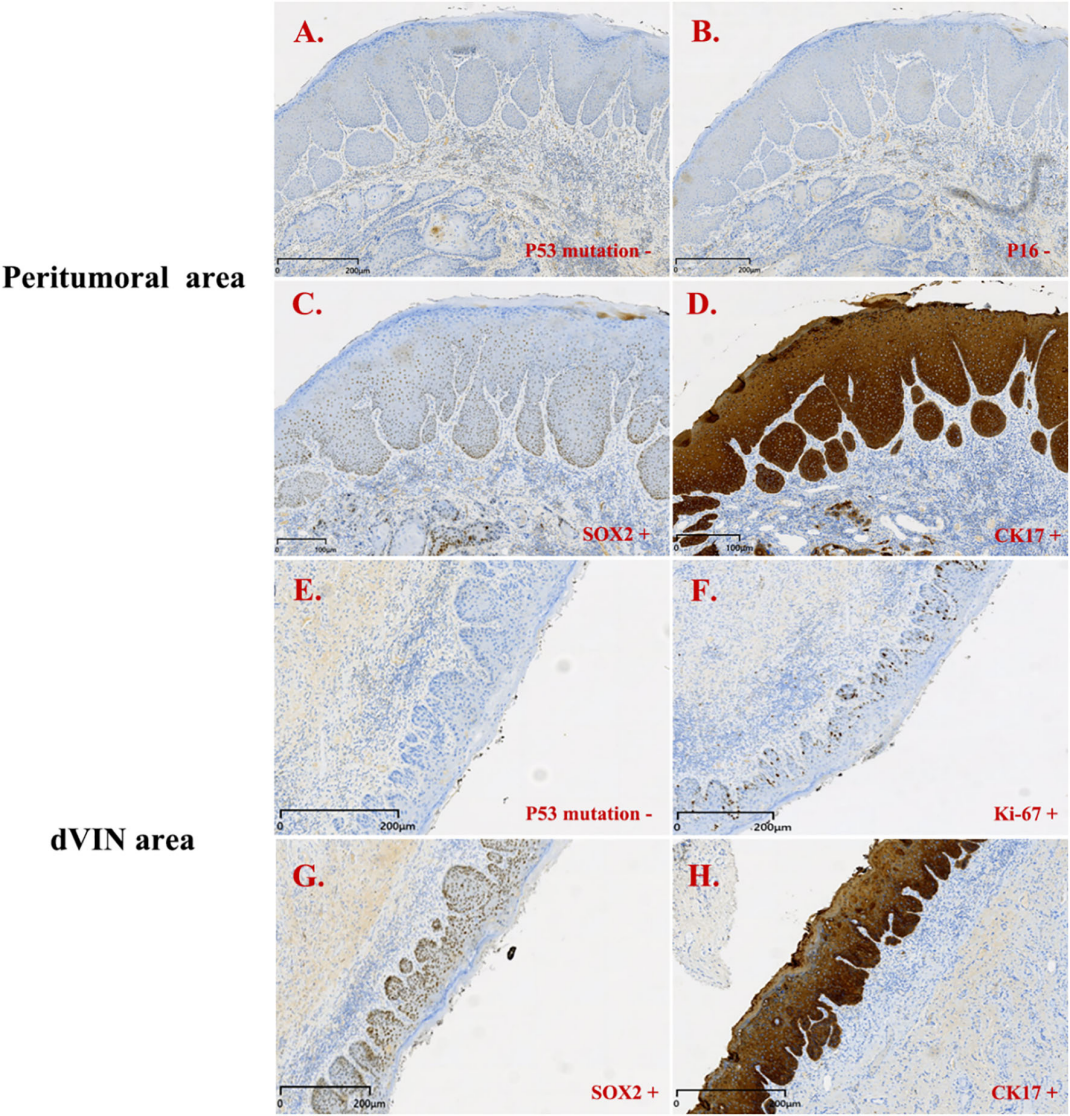
Immunohistochemistry results for patient 2. Peritumoral area (PA): **(A–D)**; dVIN area: **(E–H)**. **(A)** p53 mutation + (PA); **(B)** p16 - (PA); **(C)** SOX2 + (PA); **(D)** CK17 + (PA); **(E)** p53 mutation + (dVIN); **(F)** Ki-67 + (dVIN); **(G)** SOX2 + (dVIN); **(H)** CK17 + (dVIN).

The patient subsequently underwent vulvar mass excision and inguinofemoral lymphadenectomy. Postoperative pathology revealed HPV-independent keratinizing SCC, well- to moderately differentiated (**Figure 3**). dVIN was observed adjacent to the carcinoma, while the remaining vulvar skin exhibited features consistent with lichen sclerosus. Immunohistochemical staining of the tumor tissue revealed p16 (−), p53 (aberrant, null-type), and Ki-67 labeling index ~60%. The adjacent squamous epithelium (consistent with dVIN) showed p16 (−), p53 (aberrant, null-type), CK17 (+), SOX2 (+), and Ki-67 labeling extending to the mid-epithelial layers (~30%) (**Figure 4**).

Since the onset of the disease, the patient has not experienced postmenopausal vaginal bleeding, abdominal pain, fever, or other systemic symptoms. Both HPV and TCT (ThinPrep cytologic test) results were within normal limits. The patient subsequently received one cycle of paclitaxel plus carboplatin chemotherapy, recovered well, and had no recurrence up to the end of follow-up.

## Discussion

Both patients were middle-aged to elderly women presenting with chronic vulvar discomfort and palpable vulvar masses. During initial evaluation, both were misdiagnosed as having eczema or other inflammatory dermatoses, underscoring the ongoing challenge of distinguishing chronic vulvar lesions at an early stage. In Case 1, the patient had >10 years of vulvar pruritus with recent worsening, swelling, and pain; she was initially diagnosed with vulvar eczema at a local clinic and treated with topical anti-inflammatory agents without meaningful improvement. Only after a biopsy demonstrated squamous epithelial atypia was further investigation pursued. In Case 2, SCC was diagnosed only after lesion ulceration and biopsy.

Studies have shown that dVIN is the primary precursor lesion of HPV-independent vVSCC (10, 11). It typically arises in a background of chronic inflammatory dermatoses, especially lichen sclerosus, and often lacks specific symptoms. Clinically, dVIN may present with chronic pruritus, burning, and minor ulceration, which may mimic benign conditions such as eczema or lichen simplex chronicus, leading to delays in diagnosis and treatment (12).

The key histopathologic features of dVIN include hyperkeratosis and/or parakeratosis, acanthosis, elongated rete ridges, and prominent basal/parabasal atypia, while surface maturation is often preserved. This pattern contrasts sharply with usual-type VIN (uVIN/HSIL), which shows full-thickness epithelial immaturity; consequently, a traditional emphasis on full-thickness immaturity may lead to under-recognition of dVIN. Moreover, dVIN can overlap histologically with chronic inflammatory or lichenified lesions—particularly when epithelial hyperplasia is present—resulting in misinterpretation as a benign change (13). In our series, Case 1 was initially signed out as “Squamous cell hyperplasia (vulva) with chronic inflammation” at preliminary assessment without a clear indication of premalignant transformation, underscoring the limitations of routine H&E evaluation in dVIN diagnosis.

In this context, immunohistochemistry has become a critical adjunct for identifying dVIN and evaluating its relationship with VSCC. Multiple studies have demonstrated a high concordance between TP53 mutations in dVIN and the associated VSCC, and p53 IHC can serve as a surrogate marker for such mutations (14–17). Six p53 immunohistochemistry expression patterns have been described, of which four are considered mutation-type and two are wild-type (18, 19). Mutation-type patterns include diffuse strong overexpression and complete absence of staining (null pattern), and other abnormal distributions, and generally reflect underlying TP53 alterations. Recognition of these patterns is essential for interpreting p53 results and understanding the underlying molecular underpinnings of HPV-independent vulvar lesions. In practice, aberrant p53 expression typically presents as either diffuse, intense nuclear staining (>90%) or complete absence, with intact internal controls (null pattern), both of which are suggestive of TP53 mutation (8). Among the two patients, case 1 showed strong basal nuclear positivity (aberrant pattern), whereas case 2 exhibited complete absence of staining (null-type pattern).

In addition to p53, CK17, and SOX2 have emerged as useful diagnostic markers. Dasgupta et al. (8) found high CK17 expression in dVIN, particularly valuable in p53-wild-type cases. SOX2, a stemness-associated transcription factor, correlates with tumor cell plasticity and proliferative potential. The combined use of these markers improves diagnostic sensitivity and specificity. In our cases, both showed p53 mutant-type expression, along with positivity for CK17 and SOX2. Ki-67 is a marker of cell proliferation. In normal vulvar squamous epithelium, its expression is primarily limited to the parabasal layer, reflecting normal cellular turnover. In vulvar VIN, especially in high-grade squamous intraepithelial lesions (uVIN/HSIL), Ki-67 expression often extends upward, showing diffuse positivity in both the basal/parabasal layers, indicating increased proliferative activity. In dVIN, Ki-67 expression is typically confined to the lower one-third of the epithelium, but an expanded expression pattern may also be observed (20, 21). Therefore, an upward extension of Ki-67 staining is a crucial supportive feature for diagnosing VIN. If Ki-67 positivity is limited to the superficial layers with absent basal/parabasal labeling, the findings are atypical for VIN and are more suggestive of reactive epithelial changes (e.g., inflammatory or reparative). It is important to note that Ki-67 should not be used alone to diagnose VIN and must be interpreted in conjunction with histopathologic features and other immunohistochemical markers such as p16 and p53. In this study, Ki-67 labeling indices were approximately 40% and 60%, respectively, with positive cells mainly located in the basal to mid-epithelial layers, consistent with a typical dVIN immunophenotype. Notably, in case 2, the peritumoral epithelium also exhibited the same p53 mutant-type and SOX2 positivity, suggesting a shared molecular evolution between dVIN and the associated SCC, further supporting the role of dVIN as a critical precursor to HPV-independent VSCC.

Both patients were ultimately diagnosed with well-to moderately differentiated SCC. One had pathologically confirmed inguinal lymph-node metastasis with high metabolic activity on PET/CT (SUVmax = 14.8), compatible with metabolically active nodal metastasis despite the tumor’s well-differentiated histology. Previous studies have shown that dVIN progresses more rapidly than uVIN, with some patients developing invasive carcinoma within 6–12 months of diagnosis or presenting with VSCC at initial diagnosis (4, 22). This progression was evident in Case 1, in whom the right inguinal mass enlarged rapidly over a short period; biopsy confirmed moderately differentiated SCC with necrosis and prominent vascularity, features consistent with nodal metastasis.

With regard to treatment, both patients received radical surgical management, which included partial (wide local) vulvectomy combined with inguinofemoral lymphadenectomy. Prospective data indicate that inguinofemoral lymphadenectomy confers prognostic and survival benefits across age groups, supporting equal treatment of younger and older patients when clinically indicated (23). Given the morbidity of groin dissection (notably wound complications and up to ~30% lifetime lymphedema), current practice individualizes the extent of nodal surgery according to tumor factors and nodal risk (e.g., number/size of positive nodes, extracapsular spread, laterality), while reserving less-invasive approaches—such as sentinel lymph node biopsy (SLNB) in eligible early-stage patients (e.g., unifocal tumors <4 cm with clinically/sonographically negative groins)-to mitigate harm without compromising oncologic safety. Postoperative pathology confirmed the coexistence of dVIN and well-differentiated SCC. Prior studies indicate that timely excision of early localized lesions can significantly reduce recurrence and metastasis (24, 25). However, in cases with confirmed nodal involvement, adjuvant radiotherapy or systemic therapy may be necessary to control disease progression (26–28). More importantly, long-term and regular postoperative follow-up is essential. Pelvic and inguinal imaging is recommended every 3 to 6 months to monitor for early signs of recurrence or distant metastasis. Given the frequent coexistence of dVIN with chronic dermatologic conditions, such as lichen sclerosus, collaboration with dermatology for ongoing skin surveillance and topical steroid therapy is also recommended to prevent new lesion development. In line with contemporary surgical downsizing, eligible early-stage patients (e.g., unifocal tumors <4 cm with clinically/sonographically negative groins) may undergo wide local excision with tailored margins and SLNB rather than full inguinofemoral lymphadenectomy, with reconstruction/adjuvant therapy individualized to tumor and patient factors (29). These strategies maintain oncologic safety while reducing treatment-related morbidity and improving quality of life.

In conclusion, dVIN is a clinically subtle but high-risk precursor lesion. Its diagnosis relies heavily on histological assessment and immunohistochemical analysis. Chronic vulvar symptoms in postmenopausal women should prompt early biopsy and thorough evaluation. Our two cases illustrate the rapid progression from chronic vulvar disease to invasive carcinoma and highlight the importance of early tissue diagnosis. Future advances in molecular markers, artificial intelligence-assisted pathology, and multicenter consultations may facilitate earlier detection of dVIN and prevention of VSCC.

## Data Availability

The raw data supporting the conclusions of this article will be made available by the authors, without undue reservation.
